# Short-term hypoxic preconditioning promotes prevascularization in 3D bioprinted bone constructs with stromal vascular fraction derived cells†
†Electronic supplementary information (ESI) available: qPCR primers and Fig. S1. See DOI: 10.1039/c7ra04372d



**DOI:** 10.1039/c7ra04372d

**Published:** 2017-06-05

**Authors:** Mitchell A. Kuss, Robert Harms, Shaohua Wu, Ying Wang, Jason B. Untrauer, Mark A. Carlson, Bin Duan

**Affiliations:** a Mary & Dick Holland Regenerative Medicine Program, University of Nebraska Medical Center, Omaha, NE, USA. Email: bin.duan@unmc.edu; Tel: +1 402 559 9637; b Division of Cardiology, Department of Internal Medicine, University of Nebraska Medical Center, Omaha, NE, USA; c Division of Oral & Maxillofacial Surgery, Department of Surgery, College of Medicine, University of Nebraska Medical Center, Omaha, NE, USA; d Department of Surgery, University of Nebraska Medical Center and the VA Nebraska-Western Iowa Health Care System, Omaha, NE, USA; e Department of Surgery, College of Medicine, University of Nebraska Medical Center, Omaha, NE, USA

## Abstract

Reconstruction of complex, craniofacial bone defects often requires autogenous vascularized bone grafts, and still remains a challenge today. In order to address this issue, we isolated the stromal vascular fraction (SVF) from adipose tissues and maintained the phenotypes and the growth of endothelial lineage cells within SVF derived cells (SVFC) by incorporating an endothelial cell medium. We 3D bioprinted SVFC within our hydrogel bioinks and conditioned the constructs in either normoxia or hypoxia. We found that short-term hypoxic conditioning promoted vascularization-related gene expression, whereas long-term hypoxia impaired cell viability and vascularization. 3D bioprinted bone constructs composed of polycaprolactone/hydroxyapatite (PCL/HAp) and SVFC-laden hydrogel bioinks were then implanted into athymic mice, after conditioning in normoxic or short-term hypoxic environments, in order to determine the *in vitro* and *in vivo* vascularization and osteogenic differentiation of the constructs. Short-term hypoxic conditioning promoted microvessel formation *in vitro* and *in vivo* and promoted integration with existing host vasculature, but did not affect osteogenic differentiation of SVFC. These findings demonstrate the benefit of short-term hypoxia and the potential for utilization of SVFC and 3D bioprinting for generating prevascularized 3D bioprinted bone constructs. Furthermore, the ability to custom design complex anatomical shapes has promising applications for the regeneration of both large and small craniofacial bone defects.

## Abstract 2

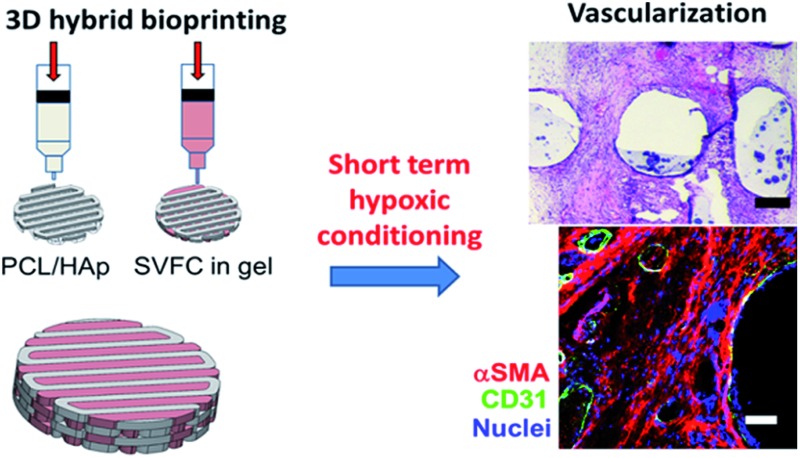
Short-term hypoxia promoted prevascularization in 3D bioprinted bone constructs with stromal vascular fraction derived cells.

## Introduction

Craniofacial fractures and bone defects, secondary to trauma, surgical resection, or congenital defects, are common causes of morbidity and generate almost 1 billion dollars of treatment costs every year in the United States.^1,2^ The repair and regeneration of craniofacial bone defects are challenging due to the complex anatomy of craniofacial structures.^3,4^ Relative success depends on the defect size, the quantity and quality of the soft tissue covering the defect, and the reconstructive approach.^5^ Currently, autogenous bone grafts are considered the gold standard.^6^ However, autogenous bone grafts are associated with certain disadvantages, such as limited availability for the reconstruction of large defects, difficulty in obtaining the required shape, and donor site morbidity.^7^ Furthermore, autogenous bone grafts have inconsistent success rates when used for reconstructing larger, critical-sized defects.^8,9^

Bone tissue engineering (BTE) is a promising technique for facilitating bone formation and regeneration by combining scaffolds, stem cells, biological factors, and physiochemical stimuli.^10,11^ However, conventional scaffold fabrication methods and regenerative strategies largely fail to reproduce both the complex 3D anatomy of craniofacial structures and the multicellular interactions present in native craniofacial tissues.^12^ Recently developed 3D bioprinting techniques have gained increased popularity in addressing some of the key challenges in craniofacial reconstruction and regeneration.^4^ 3D bioprinting implements bioinks (building blocks containing cells and/or biomaterials or cell spheroids) to produce 3D objects with complex architecture in a layer-by-layer manner.^13,14^ Importantly, 3D bioprinting also allows the fabrication of accurately shaped scaffolds/constructs by using patient imaging data (*e.g.*, magnetic resonance imaging [MRI] or computer tomography [CT]).^15,16^ 3D bioprinting enables the custom fabrication of complex craniofacial geometries with high fidelity, and supports the incorporation of cells and growth factors.^17^

Craniofacial regeneration also requires the process of neovascularization, similar to other bone regenerative processes.^18^ A lack of neovascularization within the engineered bone grafts has been shown to inhibit osteogenesis and host integration.^19,20^ Several strategies have been utilized to promote vascularization, including growth factors and other biochemical angiogenic stimulation,^21,22^ dynamic conditioning,^23^ co-culture of endothelial cells (EC) with mesenchymal stem cells (MSC),^24–26^ *in vivo* arteriovenous loops,^27,28^ and immunomodulation of adaptive immune cells.^29^ However, it is still a challenge to vascularize large craniofacial constructs. Although EC are normally used for co-culture with MSC, the former, including both human umbilical vein endothelial cells (HUVEC) and peripheral blood derived endothelial progenitor cells, have limited availability for regeneration purposes. Therefore, cell sources or cell conditioning strategies are needed to obtain an adequate amount of cells with both osteogenic and vascularization capacity within a short period of time.

Hypoxia is known to be essential for bone development through the regulation of the family of hypoxia-inducible transcription (HIF) factors.^30^ However, research results regarding the roles of hypoxia and HIF on bone regeneration and vascularization are conflicting. Some studies have shown that bone mesenchymal stem cells with hypoxia pre-treatment induced a higher degree of vascularization and enhanced osteogenesis,^31–33^ whereas other studies demonstrated that hypoxia inhibited the growth, differentiation and bone-forming capacity of osteoblasts, and osteogenic differentiation of MSC.^34,35^ This suggests that hypoxia and HIF may have multiple functions, both temporal and spatial.

In the present study, we isolated the stromal vascular fraction (SVF) from adipose tissue, and maintained the cells in a hybrid medium that supported endothelial lineage cell growth. We then 3D bioprinted polycaprolactone/hydroxyapatite (PCL/HAp) and SVF derived cell-laden hydrogel bioinks into bone constructs and conditioned the constructs in both normal oxygen and hypoxic environments for 3 weeks to assess the vascularization and osteogenic differentiation within the constructs. We also implanted the 3D bioprinted bone constructs subcutaneously into athymic mice after conditioning in normoxia *vs.* short-term hypoxia to determine the *in vivo* vascularization capacity.

## Experimental

### Stromal vascular fraction (SVF) isolation, SVF derived cell culture

SVF were isolated from subcutaneous adipose tissue of domestic pigs (*n* = 3, female, 3–4 month old), harvested immediately after euthanasia. The pigs were euthanized subjects from another research protocol, which was carried out in accordance with recommendations in the Guide for the Care and Use of Laboratory Animals from the National Research Council and the National Institutes of Health, and also in accordance with the Animal Welfare Act of the United States. The animal protocol was approved by the Institutional Animal Care and Use Committee (IACUC) of the VA Nebraska-Western Iowa Health Care System, by the IACUC of the University of Nebraska Medical Center (UNMC). All porcine procedures were performed in animal facilities approved by the Association for Assessment and Accreditation of Laboratory Animal Care International and by the Office of Laboratory Animal Welfare of the Public Health Service. The adipose tissue was carefully minced into pieces of 2–4 mm and digested in DMEM medium (Invitrogen) with 1 mg mL^–1^ collagenase type II (Worthington, Lakewood, NJ) for one hour at 37 °C. The suspension was then centrifuged at 1000 rpm for 5 min at room temperature. The mature adipocytes and supernatant were removed, and the resulting pellet was washed in phosphate buffered solution (PBS) and re-suspended in either growth medium (GM, DMEM containing 1% penicillin/streptomycin (P/S) and 10% of fetal bovine serum-FBS, Invitrogen) or hybrid medium (growth medium and EGM-2 BulletKit, Lonza, 1 : 1) after passing through a 100 μm strainer. The cells were cultured in 5% CO_2_ and 21% O_2_ in either GM or GM/EGM at 37 °C and used until passage 5.

### Flow cytometry

We removed SVF derived cells (SVFC) conditioned in GM/EGM from the culture at passage 3 by using 0.25% trypsin (Invitrogen). Cells were re-suspended in PBS, counted, then stained with Live/Dead Fixable Blue Dead Cell stain (Molecular Probes) according to manufacturer's recommendations. We then washed the cells twice with PBS and once with flow cytometry staining buffer (FCSB, 0.75% BSA, 1 mM EDTA, and 0.05% NaN_3_ in PBS). Following these washes, the cells were incubated for 15 minutes at 4 °C with mouse IgG (ChromPure, Jackson ImmunoResearch) at 1 μg per 1 × 10^5^ cells to block non-specific and Fc receptor binding. After blocking, the cells were incubated for 25 minutes at 4 °C with the following antibodies at recommended concentrations: PE CD31 (clone LCI-4, Bio-Rad), APC CD105 (clone MEM-229, Novus Biologicals), FITC CD45 (clone K252.1E4, Bio-Rad), and PE-Cy7 CD90 (clone 5E10, BioLegend). The cells were then washed 2 times with FCSB and fixed with 3% paraformaldehyde for 25 minutes at RT in the dark. Following fixation, we washed the cells 2 times with FCSB before the final resuspension in FCSB, prior to analysis. The cells were analyzed on an LSR II (Becton Dickinson) within 18 hours of staining and fixation.

### Polymer modification and hydrogel bioink preparation

Photocrosslinkable hyaluronic acid (HA, NovaMatrix, ∼1200 kDa) and gelatin (Gel, from bovine skin, Sigma) were synthesized as previously reported through the reaction of methacrylic anhydride (Sigma) in deionized water.^36^ For bioink preparation, methacrylated HA (Me-HA, 1% w/v), methacrylated Gel (Me-Gel, 4% w/v) and HA (3% w/v) were dissolved in cell culture medium with cell suspension (4 × 106 cells per ml) and 0.05% w/v 2-hydroxy-1(4-(hydroxyethox)phenyl)-2-methyl-1-propanone (Irgacure 2959; CIBA Chemicals). HA without modification was used to increase the viscosity and printability, and maintain the softness of the bioprinted constructs for cell spreading.

### Cell viability

The viability and circularity of encapsulated cells was determined using Live/Dead assay (Invitrogen) after 28 days of culture as previously described,^38^ and fluorescence images were obtained using confocal laser scanning microscopy (CLSM, LSM 710, Carl Zeiss).

### 3D bioprinting

We conducted two types of bioprinting, *i.e.* hydrogel alone and hybrid bioprinting using hydrogels and PCL/HAp. For the 3D bioprinting with hydrogel alone, the gel precursors with cells were loaded in the deposition syringes (Nordson EFD) with 22 G tips, extruded by the 3D bioprinter (3D Bioplotter, EnvisionTEC, Germany) based on the disc shaped design (*∅* 8 mm × 1 mm), and subsequently exposed to OmniCure S2000 UV lamp (Lumen Dynamics, 320 μW cm^–2^) for 45 seconds at room temperature. For bone construct bioprinting, two printing heads were used for the hybrid printing. PCL pellets (Sigma, *M*_w_ = 65 000) and HAp nanocrystals (100 nm, 10% of PCL, Berkeley Advanced Biomaterials, Inc.) were air dried at 37 °C overnight, loaded into a stainless steel syringe with a 22 G metal tip, and heated to a temperature of 140 °C in the high temperature head for 10 min. As PCL reached the molten phase, the temperature was reduced to and maintained at 120 °C during printing. A pressure of 3–3.5 bar was applied to the syringe and a deposition speed of 1.8–2.2 mm s^–1^ was used. In the low temperature head, the cell-laden gel precursors were used. These two types of materials were extruded in an alternative way to generate the whole 3D cylinder-like scaffolds (8 mm in diameter and 2 mm in thickness) designed by using Bioplotter RP and VisualMachines software (EnvisionTEC). The materials were deposited at different angles (0° and 90°) between two successive layers to create a grid pattern. The uniaxial compression testing of PCL/HAp and PCL/HAp with hydrogels was conducted using an MTS machine (INSTRON 8500 Plus, 25 KN) at a loading rate of 0.015 mm s^–1^ at room temperature. For biodegradation, 3D printed hydrogels were incubated within collagenase II (Gibco) or hyaluronidase II (Sigma) PBS solution at 37 °C under 5% CO_2_ for up to 21 days. At each predetermined time point, the weight of hydrogel discs were measured, and changes of weight were normalized to their initial weight before degradation.

The cell laden constructs were washed with PBS and maintained at 37 °C in either normoxic (5% CO_2_, 21% O_2_) or hypoxic (5% CO_2_, 21% O_2_, in Tri-gas incubator, Thermo Fisher Scientific) conditions. The hydrogel-alone constructs were conditioned in GM/EGM medium for up to 14 days. The 3D bioprinted bone constructs were conditioned in hybrid medium with osteogenic medium (containing GM, 100 nM dexamethasone, Sigma; 10 mM β-glycerophosphate, Sigma; and 50 μM ascorbic acid, Sigma) and EGM (OGM/EGM) for 21 days.

### Alkaline phosphatase (ALP) staining and activity

Alkaline phosphatase leukocyte kit (Sigma) was used according to the manufacturer's protocol for ALP staining. ALP activity quantification was performed as previously described.^36^ Briefly, the lysed cells from 3D bioprinted constructs were mixed with ALP substrate solution containing *p*-nitrophenyl phosphate (pNPP) (Sigma) at 37 °C for 25 min. The reaction was stopped by the addition of NaOH, then the production of *p*-nitrophenol in the presence of ALPase was measured by monitoring the absorbance of the solution at a wavelength of 405 nm using a microplate reader (Bio-Tek Instruments). The total protein content was determined using a BCA assay kit (Pierce, Rockford, IL, USA) with bovine serum albumin as a standard, and the ALP activity was expressed as μmol of *p*-nitrophenol formation per minute per milligram of total proteins (μmol per min per mg protein).

### RNA isolation and quantitative real time polymerase chain reaction (qPCR)

For qPCR analysis, the samples were first homogenized in lysis buffer using a bead miller (Fisher Scientific). Total RNA was extracted from cell-laden constructs using QIA-Shredder and RNeasy mini-kits (QIAgen) according to the manufacturers' instructions. Total RNA was synthesized into first strand cDNA in a 20 μL reaction using an iScript cDNA synthesis kit (BioRad Laboratories). Real-time PCR analysis was performed in a StepOnePlus™ Real-Time PCR System (Thermo Scientific) using SsoAdvanced SYBR Green Supermix (Bio-Rad). cDNA samples were analyzed for the gene of interest and for the housekeeping gene 18S rRNA. The level of expression of each target gene was calculated using the comparative Ct (2^–ΔΔCt^) method.^36,37^ The designed primers are listed in ESI Table S1.†

### *In vivo* subcutaneous implantation

Eight-week old, female athymic nude mice from Jackson Laboratory were used. The mice were housed in compliance with UNMC guidelines. All surgery procedures were reviewed and approved by the IACUC of UNMC. The cell laden bone constructs were conditioned in hybrid medium (OGM/EGM) for 21 days, then five samples of each group were subcutaneously implanted in the nude mice (two samples per mouse and 6 mice in total). For the surgery, the mice were deeply anesthetized and one dorsal incision was made, lateral to the spine. Two subcutaneous pockets were made from this single incision, one on each side of the mouse, using blunt dissection. The scaffold implants were gently inserted into the subcutaneous pockets. The single skin incision was closed with wound clips and mice were monitored closely to ensure full recovery from anesthesia. All of the animals were sacrificed after 4 week implantation.

### Histological and immunohistochemical (IHC) staining

2D cultured SVFC and cell-laden hydrogels were fixed in 4% paraformaldehyde, and printed PCL/HAp based bone constructs were fixed in 10% neutralized formalin overnight. The PCL/HAp based bone constructs were embedded in optimal cutting temperature (OCT) compound and cross-sectioned (∼10 μm thick) using a cryosectioner (Leica). H&E staining was conducted for implanted scaffolds. For immunohistochemical staining, the samples (cells, hydrogels, or sectioned slides) were permeabilized in 0.2% Triton X-100 and then blocked with 1% bovine serum albumin (BSA) overnight at 4 °C. The samples were incubated with primary antibodies to α-smooth muscle actin (αSMA) (1 : 200, Sigma), anti-CD31 (1 : 100, Cell Signaling) and von Willebrand factor (vWF, 1 : 100, Sigma) overnight at 4 °C. Secondary fluorescent antibodies and/or Alexa Fluor 488-conjugated phalloidin (1 : 40, Invitrogen) were incubated for 2 h, and nuclear counterstaining (Draq 5, 1 : 1000, Thermo Scientific) was performed for 30 minutes at room temperature. The stained samples were imaged with Zeiss 710 CLSM.

### Quantitative estimation of microvessel density and area distribution

Microvessel density and area within the constructs after *in vivo* implantation were calculated by using two images per sample from the images of five scaffold samples (totally, 10 images for each group). The microvessels were identified as luminal structures with CD31 positive cells.

### Statistical analysis

All quantitative data was expressed as mean ± standard deviation (SD). Pairwise comparisons between groups were conducted using ANOVA with Scheffé post-hoc tests. A *p*-value of <0.05 was considered statistically significant.

## Results

### Maintenance of endothelial lineage cells within SVFC

**Fig. 1:**
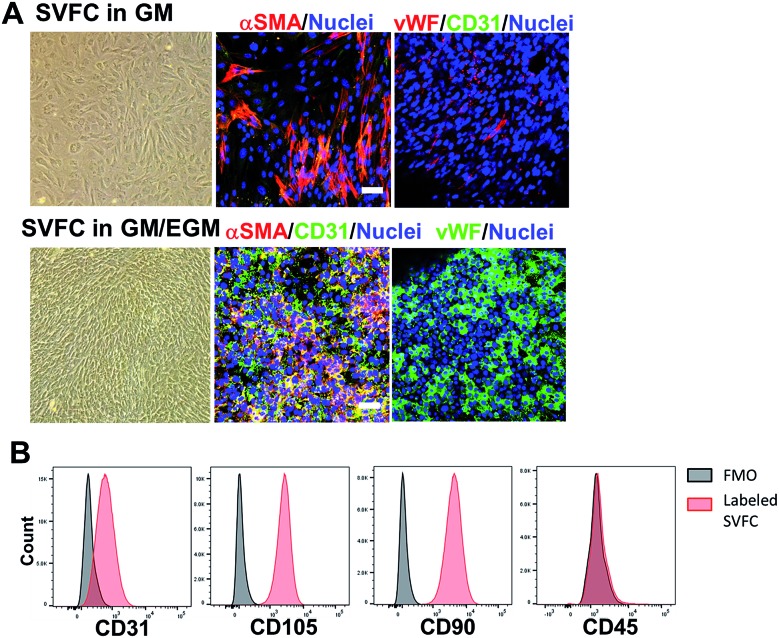
The incorporation of EGM helped the maintenance of endothelial lineage cells and their phenotypes within SVFC. (A) 2D culture of SVFC in GM or GM/EGM. In GM, SVFC showed a spindle-like morphology, expressed αSMA and were negative for CD31 and vWF. SVFC expressed αSMA, CD31 and vWF in the culture with addition of EGM-2 medium (scale bar = 50 μm); (B) the expression of CD31, CD105, CD90 and CD45 by SVFC in GM/EGM was examined by flow cytometry. Grey histograms represent fluorescence minus one (FMO) controls, while red histograms are from SVFC that have been labelled as indicated. Graphs for CD105, CD90, and CD31 are derived from living, singlet, CD45-events. The graph for CD45 is from living, singlet events. SVFC were positive to CD31, CD105, CD90, but negative to CD45.

We first isolated the SVF from porcine adipose tissue, then we conditioned the SVFC in either DMEM based GM or hybrid medium with GM and endothelial medium (EGM). In GM, the SVFC demonstrated a spindle-like morphology, had strong expression of αSMA, with limited expression of vWF and CD31 (Fig. 1A). In contrast, when SVFC was conditioned in GM/EGM, they were positive for both stromal cell markers (αSMA) and endothelial lineage cell markers (CD31 and vWF) (Fig. 1A). This data suggested that the addition of EGM-2 medium supported the maintenance of endothelial lineage cells within SVF. The flow cytometry results demonstrated that SVFC in GM/EGM were CD105, CD90, and CD31 positive, and CD45 negative (Fig. 1B). This suggested that SVFC were a heterogeneous cell population with stromal cells and endothelial cells, but not hematopoietic cells.

### Short-term hypoxic conditioning promoted vascularization, whereas long-term condition impaired cell viability in GM/EGM

**Fig. 2:**
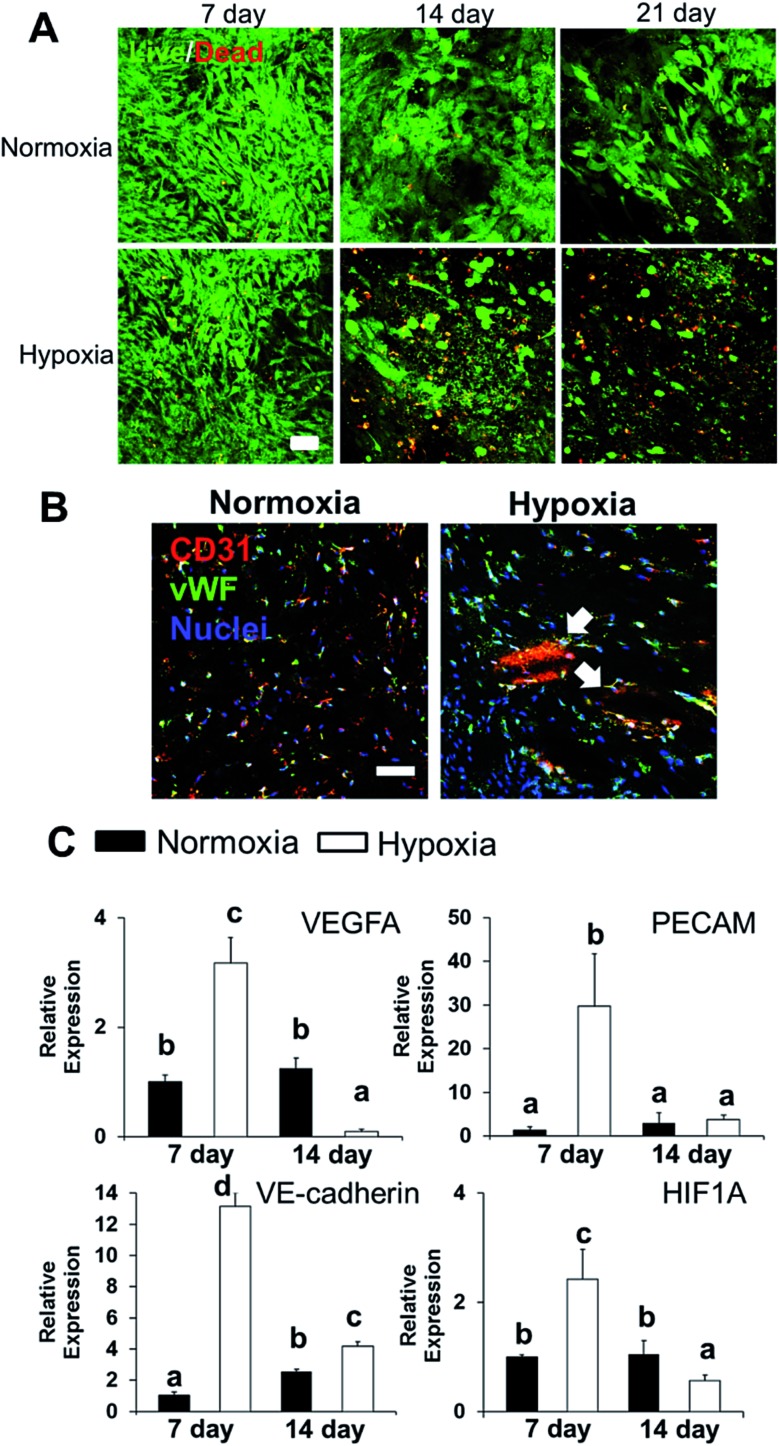
Effects of hypoxia on SVFC viability and vascularization. (A) Long-term hypoxic conditioning significantly affects SVFC viability. Cell viability for the 3D bioprinted hydrogel constructs with SVFC conditioned in GM/EGM in either hypoxia or normoxia for up to 21 days (scale bar = 50 μm); (B) IHC staining of SVFC laden constructs showed that SVFC were positive for both CD31 and vWF in both normoxia and hypoxia after 7 day culture. In hypoxia, microvessel-like structures were also observed, indicated by white arrows (scale bar = 50 μm); (C) qPCR analysis of VEGFA, PECAM1, VE-cadherin and HIF1A in SVFC laden hydrogels after 7 and 14 day culture in GM/EGM in normoxia and hypoxia. Relative gene expression is presented as normalized to 18S and expressed relative to cells in normoxia for 7 days (*n* = 3; bars that do not share letters are significantly different from each other; *p* < 0.05).

We conditioned 3D bioprinted SVFC-laden hydrogel constructs in GM/EGM, then subjected the constructs to either normoxia or hypoxia for up to 21 days. At day 7, SVFC showed high cell viability in both normoxic and hypoxic environments (Fig. 2A). However, at 14 and 21 days of hypoxia, increasing numbers of dead cells were found in the 3D bioprinted hydrogel constructs (Fig. 2A). IHC staining images showed that SVFC within 3D bioprinted constructs were positive for CD31 and vWF in both normoxia and hypoxia (Fig. 2B). The encapsulated SVFC also formed microvessel-like structures within the constructs conditioned in hypoxia (Fig. 2B indicated by white arrow). QPCR analysis results confirmed that at day 7, the cells in the hypoxic environment significantly upregulated vascularization related gene expression (*i.e.* VEGFA, PECAM, VE-cadherin, and HIF1A). At day 14, however, the gene expression in the hypoxia group was downregulated compared to day 7, and some expression was even lower than the normoxia group (Fig. 2C).

### 3D hybrid bioprinting of bone constructs

**Fig. 3:**
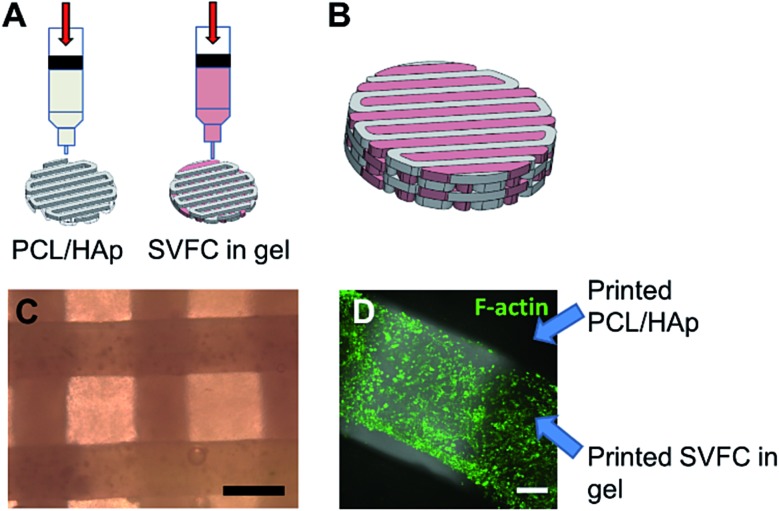
3D hybrid bioprinting of SVFC laden bone constructs. (A) Schematic of 3D hybrid bioprinting using multiple cartridges. Multiple PCL/HAp frames were first printed using high temperature printing cartridge in each layer throughout the construct, then SVFC and hydrogels were deposited in between the frames by using a low temperature cartridge; (B) model of bioprinted bone constructs; (C) bright field image showed the part of bioprinted PCL/HAp and hydrogel construct (scale bar = 500 μm); (D) IHC staining of F-actin showed that the SVFC laden hydrogel was sandwiched between the two frames of PCL/HAp (scale bar = 100 μm).

We integrated the SVFC-laden hydrogel bioink together with PCL/HAp to create 3D hybrid bioprinted bone constructs. These two bioinks were printed in an alternate order. PCL/HAp was printed first, followed by bioprinting of the cell-laden hydrogel in the groove created by the first material on the same layer (Fig. 3A). Then, layer-by-layer, we printed the constructs with cells inside (Fig. 3B and S1†). The bright field microscopic image showed that the bioprinting of these two bioinks was even (Fig. 3C). IHC staining with F-actin showed that the encapsulated SVFC were between the PCL/HAp scaffolds (Fig. 3D).

**Fig. 4:**
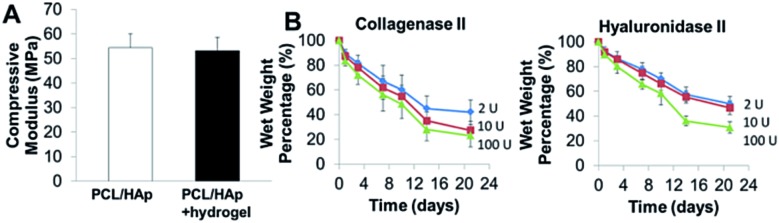
(A) Compressive modulus of 3D printed PCL/HAp and hybrid printed PCL/HAp and hydrogel scaffolds; (B) biodegradation of bioprinted hydrogels in collagenase II or hyaluronidase II solution.

Compressive tests were conducted for PCL/HAp scaffolds with and without hydrogels, as shown in Fig. 4A. The compressive modulus of the PCL/HAp scaffold was slightly higher than that of hybrid bioprinted scaffolds, but did meet the level of statistical significance. PCL has a relatively long degradation time (around 1.5 to 2 years).^38^ This provides long-term structural stability and avoids the accumulation of acidic degradation products. However, both Me-HA and Me-Gel based hydrogels are degradable within a relatively short time, especially within a cellular environment. We thus measured the degradability of bioprinted Me-HA/Me-Gel hydrogels instead of the whole hybrid bioprinted scaffolds. Without enzymes, the printed hydrogels had very limited degradation over 21 day incubation. In the solution with collagenase II or hyaluronidase II, the hydrogels degraded faster with increasing higher enzyme concentration (Fig. 4B). In addition, it was found that collagenase II promoted more hydrogel degradation compared to hyaluronidase II with the same concentration. This is probably because there was more Me-Gel in the hydrogel, and Me-Gel has smaller molecular weight compared to Me-HA.

### Short-term hypoxic conditioning promoted vascularization, but did not affect osteogenic differentiation of SVFC laden bone constructs in OGM/EGM

**Fig. 5:**
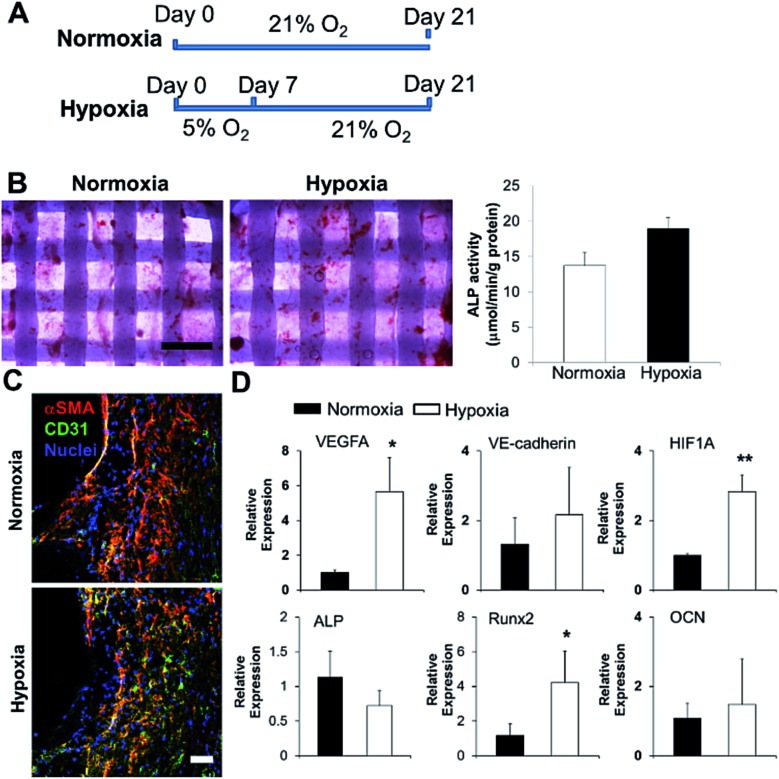
Effects of short-term hypoxic treatment on SVFC osteogenic differentiation and vascularization within 3D bioprinted constructs in OGM/EGM. (A) Timeline and treatment for 3D bioprinted SVFC laden constructs. Hypoxia group represented the 7 day hypoxic conditioning and 14 day normoxic conditioning, while the normoxia group represented 21 day culture in normal oxygen tension; (B) short-term hypoxia did not significantly change ALP expression and activity (scale bar = 1 mm); (C) IHC staining of αSMA and CD31 after in total 21 day culture in both groups (scale bar = 50 μm); (D) qPCR analysis of vascularization markers (*i.e.* VEGFA, VE-cadherin and HIF1A) and osteogenic differentiation markers (*i.e.* ALP, Runx2 and OCN) in 3D bioprinted constructs after 21 day culture in normoxia and hypoxia groups. Relative gene expression is presented as normalized to 18S and expressed relative to cells in normoxia for 21 days (*n* = 3; *p < 0.05, **p < 0.01).

We 3D bioprinted bone constructs using SVFC-laden hydrogels and PCL/HAp, conditioned the constructs under hypoxia in OGM/EGM for 7 days, and subsequently conditioned them in normoxia for another 14 days (Fig. 5A). The bone constructs in the normoxic environment for 21 days served as a control. We compared the osteogenic differentiation ability of SVFC to determine whether short-term hypoxic conditioning affected osteogenic differentiation *in vitro*. The 3D bioprinted constructs were stained positive for ALP both in normoxia and hypoxia (Fig. 5B). The hypoxia group showed slightly higher ALP activity compared to the normoxia group after 21 day of culture, but this difference was not statistically significant (Fig. 5B). PCR results showed that Runx2 expression was upregulated in the hypoxia group, but no statistical difference was observed for ALP and OCN expression (Fig. 5D). These results indicate that 7 day hypoxic conditioning did not affect the *in vitro* osteogenic differentiation of SVFC. SVFC-laden constructs were positive for CD31 and αSMA in both normoxic and short-term hypoxic environments, as displayed in the IHC staining images (Fig. 5C). In hypoxia, the expression of CD31 was more obvious (Fig. 5C). QPCR results confirmed that short-term hypoxia promoted vascularization of SVFC in OGM/EGM by significantly upregulating VEGFA and HIF1A expression (Fig. 5D).

### Short-term hypoxic conditioning enhanced *in vivo* formation of vascular networks

**Fig. 6:**
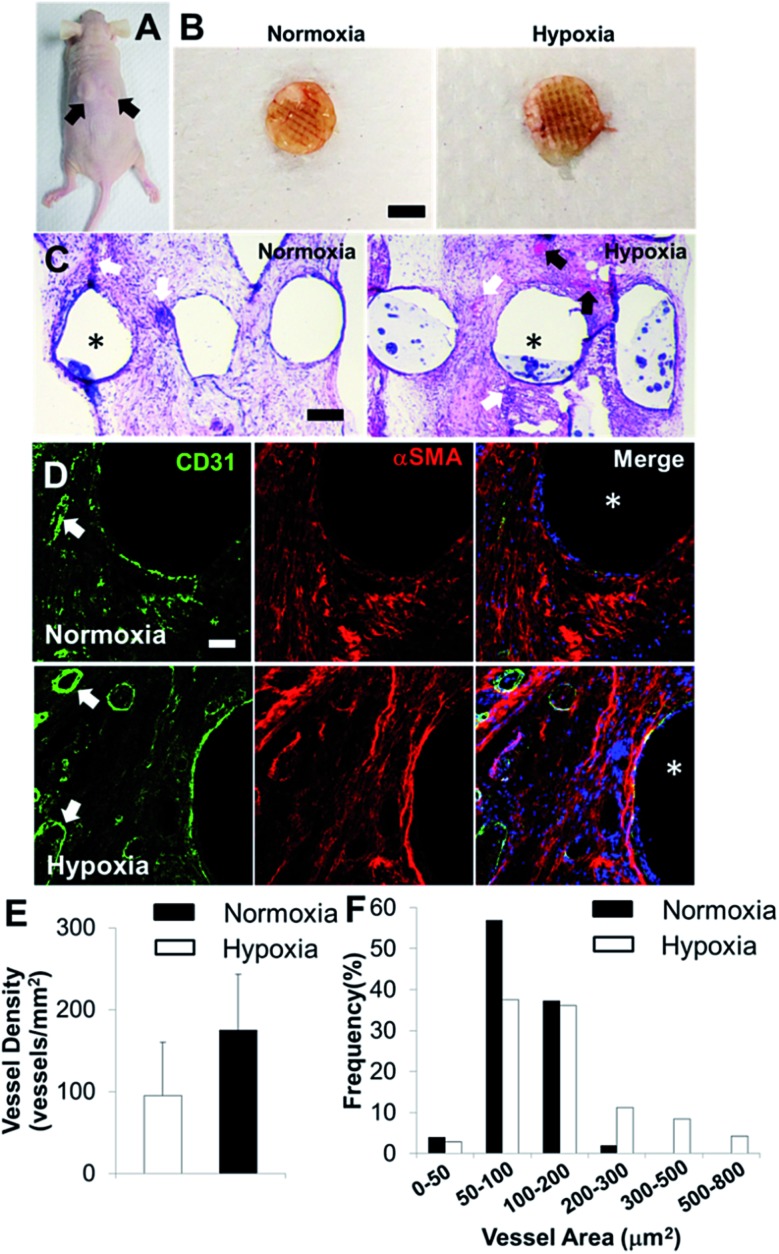
*In vivo* implantation and vascularization within 3D bioprinted bone constructs. (A) Two bioprinted constructs after 21 day *in vitro* culture were subcutaneously implanted in the athymic mouse (as indicated by black arrows); (B) overall view of explanted constructs after 4 week subcutaneous implantation (scale bar: 5 mm); (C) representative images of H&E staining (scale bar: 200 μm); (D) IHC staining for CD31 (green), αSMA (red), and nuclei (blue) within 3D bioprinted constructs (scale bar: 50 μm). The white arrows indicate the formed microvessels with lumen structures, and the black arrows indicate the murine erythrocytes within blood vessels. The asterisks indicate the PCL/HAp scaffold strand areas; quantification of CD31 positive vascular-network within 3D bioprinted constructs in hypoxia and normoxia groups by measuring the microvessel density (E) and microvessel area distribution (F) (two images per sample from the images of five scaffold samples, 10 images in total).

In order to determine the capacity of the vascular network formation within 3D bioprinted bone constructs *in vivo*, we surgically implanted cell-laden constructs into immunodeficient mice for 4 weeks. The 3D bioprinted SVFC-laden constructs were conditioned either in a short-term hypoxic environment (7 day hypoxia and 14 day normoxia as shown in Fig. 5A) or in a normoxic environment for 21 days in hybrid media consisting of OGM and EGM before implantation. Fig. 6A shows the athymic mouse after the construct implantation into skin pockets on both sides. None of the constructs evoked any noticeable host inflammatory response during 4 week implantation. Fig. 6B shows macroscopic views of constructs in short-term hypoxia and normoxia after explanation. The constructs in both groups showed obvious blood vessels along the channel areas. H&E staining in Fig. 6C show that the 3D bioprinted constructs were densely populated with cells (both host mouse cells and encapsulated SVFC) throughout the constructs. The constructs in both groups showed obvious microvascularity. However, more obvious murine erythrocytes (black arrows, Fig. 6C) were also observed within the microvessels in the constructs conditioned in the hypoxic environment, indicating the integration of formed lumens with existing host vasculature. The perfused microvasculature stained positive for CD31 in the constructs in both normoxia and hypoxia (Fig. 6D). The CD31 positive vessel density in short-term the hypoxia group was slightly higher than that in the normoxia group, without significant difference (Fig. 5E). In the hypoxia group, both the median and average lumen sizes (120.3 ± 67.7 μm^2^ and 226.6.0 ± 73.8 μm^2^) were significantly larger than those in the normoxia group (82.8 ± 56.2 μm^2^ and 106.7 ± 62.8 μm^2^), and the vessel area distribution was also broader (Fig. 6F).

## Discussion

Reconstructing the complex anatomy of craniofacial defects resulting from trauma, cancer resection, and congenital malformations, still remains a clinical challenge. Although several strategies have been explored to generate 3D scaffolds and promote vascularization in them, there is still a significant clinical need to develop scaffold fabrication techniques and to identify more clinically relevant cell sources with both osteogenic and vascularization capacity. In our current study, we implemented SVF derived from adipose tissues as a cell source, and maintained stromal cells and endothelial lineage cells within SVFC. We further 3D hybrid bioprinted bone constructs by using SVFC-laden, bioactive hydrogels consisting of Me-HA, Me-Gel, and PCL/HAp.

Adipose tissue represents an attractive source for tissue regeneration due to its abundance, surgical accessibility, and content of a heterogeneous cell population, including adipocytes, multipotential stromal cells, and endothelial progenitor cells.^39,40^ The mesodermal cell population, defined as SVF, is typically harvested by enzymatic digestion and centrifugation, and contains a heterogeneous array of cell types, including mesenchymal stem cells, endothelial cells, perivascular cells, fibroblasts, and immune cells.^41,42^ SVF has gained increased popularity and has wide applicability in the regeneration of the spinal cord,^43^ intervertebral disc,^44^ and cartilage.^45,46^ However, most SVF were directly injected into the target tissues or generated into pellets for implantation without *in vitro* expansion. These strategies may limit broader applications of SVF in many other tissue regeneration processes, where a large number of cells are needed. In this study, we maintained the SVFC in either GM or GM/EGM, instead of direct use. Our results demonstrated that the addition of EGM helped with the maintenance of endothelial lineage/progenitor cells, and the whole heterogeneous cell population contained functional stromal cells and endothelial cells, however, it lacked hematopoietic cells. This simple strategy represents a more suitable method for a one-step surgical procedure to provide much more SVFC within short time.

We then used SVFC in the hybrid medium of GM/EGM for 3D bioprinting. We conditioned the 3D bioprinted SVFC laden hydrogel constructs in either a normoxic or a hypoxic environment for up to 21 days. We found short-term (up to 7 days) hypoxia promoted vascularization of SVFC laden constructs without significantly affecting cell viability, whereas long-term (more than 14 days) hypoxia impaired the cell viability and vascularization. These results indicate that the effects of hypoxia are spatial and temporal, depending on the stage of bone repair/regeneration. These results may also be partially explained by current conflicting studies about the role of hypoxia on vascularization. At the early stage of bone repair or regeneration, despite the increase in blood flow in response to the injured extremity, a period of hypoxia follows, which is a normal process during healing.^47^ This short-term period of hypoxia promotes the vascularization in later stages by promoting secretion of various factors, including the vascular endothelial growth factor (VEGF) and interleukin-6 (IL-6).^48^ Similar effects were also reported by other studies. For example, hypoxic pre-treatment of bone marrow mesenchymal stem cells facilitated angiogenesis by improving the function of endothelial cells in diabetic rats with lower ischemia.^49^ In our current study, we did not further optimize the hypoxia treatment time. It is possible that shorter (less than 7 days) or even transient hypoxia has maximum effects on SVFC vascularization capacity.

3D printing is a particularly promising technology for bone tissue engineering,^50,51^ which enables the creation of 3D scaffolds with controllable pore size, porosity, and internal architecture in a layer-by-layer manner.^52,53^ More evidence has shown that the lack of vascularization within the engineered bone grafts have impaired osteogenesis and host integration. Wang *et al.* 3D printed porous poly(propylene fumarate) (PPF) scaffolds and further implanted them into a rat model, which demonstrated that vascularization decreases with increasing pore size. In our bioprinting strategy, dual bioprinting cartridges were used to create multiple PCL/HAp frames in each layer throughout the construct then deposit SVFC and hydrogels in between the frames. The 3D bioprinted bone constructs had sufficient mechanical strength due to the abundance of uniformly distributed PCL/HAp frames and integrated cellular components. We designed the pore size between PCL/HAp frames to be ∼400 μm, and the covered hydrogels can also serve as a vascular bed to support vascularization. Mishra and co-workers developed a composite scaffold system of a 3D printed PPF shell and fibrin hydrogel with an MSC/HUVEC spheroid to assist vascular infusion.^26^ Kang *et al.* demonstrated that the bioprinted calvarial bone constructs with human amniotic fluid-derived stem cells (AFSC) promoted bone and vessel formation.^13^ In our current study, we implemented SVFC which are more available and clinically relevant. Mao's group integrated Arg-Gly-Asp (RGD)-phage nanofibers into the pores of 3D printed, biphasic calcium phosphate scaffolds to induce the regeneration of vascularized bone *in vivo*.^54^ Our 3D bioprinted composite scaffolds could also promote superior *in vivo* microvessel formation in the whole construct. This bioprinting strategy enables fabrication of clinically relevant sized tissue constructs with complex architecture and cellular integrity for craniofacial regeneration. In order to demonstrate the capacity of hybrid bioprinting, we reconstructed skull CT images of one patient with a craniofacial defect, and reconstructed the defect site on the lower jaw. Based on the reconstructed defect geometry, we 3D printed the construct with the patient specific structure by using PCL/HAp and hydrogel (Fig. S1†).

The 3D bioprinted bone constructs were conditioned in hypoxia for 7 days, then in normoxia for another 14 days in OGM/EGM. The effects of hypoxia on osteogenic differentiation of MSC were also conflicting. Yang *et al.* reported that hypoxia inhibited osteogenesis in human MSC through direct regulation of Runx2 by Twist.^55^ In contrast, Valorani *et al.* demonstrated that pre-culturing human adipose derived MSC under hypoxia increased their adipogenic and osteogenic differentiation potentials.^56^ We found that short-term hypoxic treatment did not affect osteogenic capacity of SVFC in the constructs compared to 21 day culture in normoxia. The inconsistent results may also be due to the applied hypoxia degree and MSC sources. It has been reported that the development of vascularization prior to osteogenesis could better promote osteogenic induction. Our results demonstrated that osteogenic differentiation and vascularization of SVFC within 3D bioprinted constructs co-occurred due to the cellular heterogeneity. Further effort should be made to synergize vascular development and osteogenesis in the temporal manner.

The 3D bioprinted bone constructs with SVFC alone supported *in vivo* vascularization in the subcutaneous mice model. As previously mentioned, SVFC were heterogeneous cell populations with not only mesenchymal stromal cells and endothelial cells, but also smooth muscle cells and pericytes. Direct contact between endothelial cells and all of the stromal cells is essential for mature vascular network formation, as the stromal cells, especially pericyte like cells, expressed the pericyte marker to stabilize the formed vessels.^57^ The short-term hypoxic conditioning promoted vascularization both *in vitro* and *in vivo*, partially, by upregulating VEGF and HIF1A expression. Although the vessel density in the short-term hypoxia group did not significantly exceed their normoxia counterpart, the median and average lumen sizes and size distribution in hypoxia group were statistically larger and broader than those in the normoxia group. This indicated that short-term hypoxia promoted the microvessel growth and stabilization. The hybrid 3D bioprinting also enables the incorporation of growth factors like VEGF and/or growth factor delivery systems like nano-/microparticles for further facilitating the vascularization *in vitro* and *in vivo*.

## Conclusions

In summary, our current study expanded SVFC isolated from adipose tissue and maintained the endothelial lineage cells by the incorporation of EGM. We encapsulated SVFC within 3D bioprintable hydrogel bioinks in either normoxia or hypoxia. We found that short-term hypoxic conditioning promoted vascularization related gene expression, whereas long-term hypoxia impaired cell viability and vascularization. We further 3D bioprinted composite scaffolds consisting of PCL/HAp frames and SVFC laden hydrogels for the prevascularization of engineered bone constructs. The short-term hypoxic conditioning promoted microvessel formation *in vitro* and *in vivo*, along with integration with existing host vasculature, but did not affect osteogenic differentiation of SVFC. The SVFC are promising cell sources for craniofacial bone regeneration, and our hybrid bioprinting strategy allows the generation of 3D anatomical shapes with multiple types of cells and biomaterials. Short-term hypoxic treatment also promotes the prevascularization of bioprinted bone constructs *in vitro* to achieve rapid anastomosis *in vivo* and enhances bone repair.
