# Supplementary material for: Short-term hypoxic preconditioning promotes prevascularization in 3D bioprinted bone constructs with stromal vascular fraction derived cells
Source: RSC Adv. 2017 Jun 5;7(47):29312–20. doi: 10.1039/c7ra04372d (PMC5472052; doi:10.1039/c7ra04372d)
Supplement: Supplementary file 1 [file RA-007-C7RA04372D-s001.pdf]

Electronic Supplementary Material (ESI) for RSC Advances.
This journal is © The Royal Society of Chemistry 2017

Support information

Short-Term Hypoxic Preconditioning Promotes Prevascularization in 3D Bioprinted Bone Constructs with Stromal Vascular Fraction Derived Cells

Mitchell A. Kuss,[ab] Robert Harms,[ae] Shaohua Wu,[ab] Ying Wang,[ab] Jason B. Untrauer,[c] Mark A. Carlson[ad] and Bin Duan*[abe]

[a]Mary & Dick Holland Regenerative Medicine Program, University of Nebraska Medical Center, Omaha, NE, USA

[b]Division of Cardiology, Department of Internal Medicine, University of Nebraska Medical Center, Omaha, NE, USA

[c]Division of Oral & Maxillofacial Surgery, Department of Surgery, College of Medicine, University of Nebraska Medical Center, Omaha, NE, USA

[d]Department of Surgery, University of Nebraska Medical Center and the VA Nebraska-Western Iowa Health Care System, Omaha, NE, USA

[e]Department of Surgery, College of Medicine, University of Nebraska Medical Center, Omaha, NE, USA

*Corresponding author: E-mail address: bin.duan@unmc.edu; Phone: (402) 559-9637

Supplemental Table S1 Real-Time PCR Primers

Gene symbol	Genbank ID	Primer sequences (5'→3')	Product size (bp)
18S	NR_003286	F:GAGAAACGGCTACCACATCC<br>R: CACCAGACTTGCCCTCCA	170
VEGFA	NM_001025366.2	F: CTGGAGCGTGTACGTTGGT<br>R: TGCAACGCGAGTCTGTGTTT	155
PECAM1	NM_000442.4	F: CCACGCCTAGCCAAAATCAC<br>R:CATGTGGCCCCTCAGAAGAC	136
VE-cadherin	NM_001795.4	F:ATGAGATCGTGGTGGAAGCG<br>R: TGTGTACTTGGTCTGGGTGA	125
HIF1A	NM_001530.3	F: ATCACCCTCTTCGTCGCTTC<br>R: GAAAGGCAAGTCCAGAGGT	164
ALP	NM_000478	F: CCACAAGCCCGTGACAGA<br>R: GGGCGGCAGACTTTGGTT	127
Runx2	NM_001024630	F: TACCTGAGCCAGATGACG<br>R: AAGGCCAGAGGCAGAAGT	145
OCN	NM_199173	F: GGCAGCGAGGTAGTGAAGA<br>R: CCTGAAAGCCGATGTGGT	148

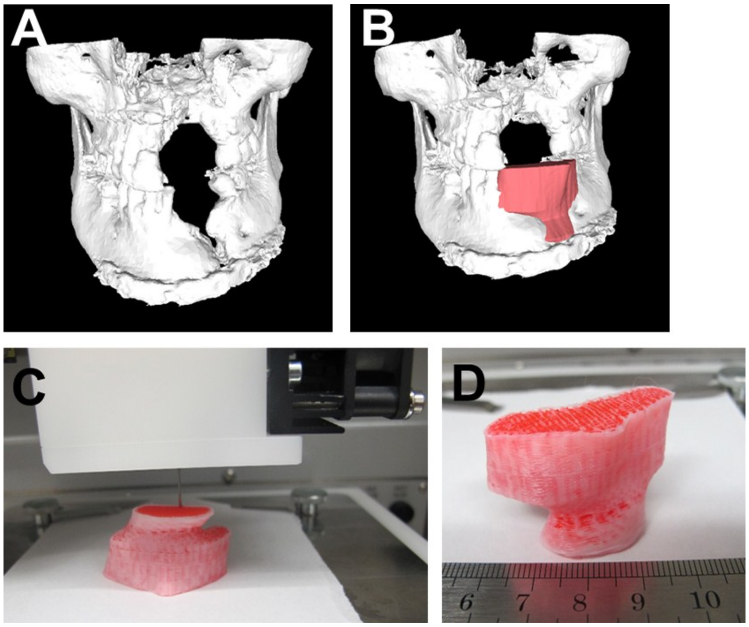

Figure S1. 3D hybrid printed human sized mandibular bone construct. (A) Reconstructed skull model from a patient with facial defect; (B) reconstruction of lower jaw bone; (C) 3D hybrid printing of the reconstructed lower jaw bone; (D) 3D printed bone construct with human size and anatomical architecture.
